# P09 Lancashire and South Cumbria Critical Care Network Vancomycin Project

**DOI:** 10.1093/jacamr/dlad143.013

**Published:** 2024-01-03

**Authors:** Shaun Morgan, Kelly Newby, Hisham Ziglam

**Affiliations:** Lancashire and South Cumbria Critical Care Network; Furness General Hospital; University Hospitals Morecambe Bay; East Lancashire Hospitals NHS Trust

## Abstract

**Background:**

This project was a collaborative piece of work across a critical care network that included specialist advice from colleagues including aseptic/technically trained pharmacists and microbiology to optimize care for critical care patients in Lancashire and South Cumbria. We used several members of our workforce including pharmacists, nursing staff, microbiologists, rotational doctors and consultants to work towards a harmonized guideline.

**Methods:**

During our initial meeting, we identified that different centres were using different glycopeptides and administering them differently: East Lancashire Hospitals (ELHT)—continuous vancomycin infusion; Blackpool Cardiac and Blackpool General—continuous vancomycin infusion, with no formal guideline in place but adopted from ELHT; Lancashire Teaching Hospitals—intermittent vancomycin infusions; and University Hospitals Morecambe Bay—teicoplanin is glycopeptide of choice. Our consultant microbiologist discussed the use of glycopeptides and what infections they are used for in critical care. The pharmacokinetics of vancomycin and the benefit of using a continuous infusion was also discussed. We decided to undertake a clinical audit of our vancomycin guidelines at ELHT to ensure that we had an evidence base to implement our guideline and provide assurance to the working group. We agreed to reconvene the working group after this audit was completed and after the concerns of colleagues had been addressed.

**Results and conclusions:**

We presented the results of our audit and highlighted the opportunities harmonizing this guideline would provide. We discussed various scenarios such as stepping up from a ward protocol to a continuous infusion and the different methods of calculating creatinine clearance and if this would influence the first 24 h of the infusion. One comment was concerning the administration of vancomycin and the number of times nurses will be changing bags for patients requiring higher doses, i.e. the majority of patients would usually be on 3 g/day or more depending on concentrations. This would mean changing the bag more than twice a day; as much as there is a therapeutic benefit to continuous vancomycin infusions, considering the max. concentration for peripheral and central lines allows for larger volumes, it would be beneficial (except in fluid restricted cases) to have a slightly larger volume to reduce the number of bag changes and plastic consumption. We considered this comment and discussed it with our aseptic services lead pharmacist. We attended a meeting of the ICS Aseptics Working Party across Lancashire and South Cumbria. WE discussed the proposed guideline and requested that aseptic units across Lancashire and South Cumbria developed worksheets to manufacture 5 g in 600 mL or 10 g in 1.2 L for central use to stop breaking the line when changing a bag and reducing plastic consumption. We also met with critical care practice educators across the network to discuss how we can promote this guideline and ensure nursing staff and prescribers are aware of how to prescribe and administer continuous vancomycin infusions. One of our units noted that they have had a few incidents with incorrect concentrations of vancomycin being made up for patients. We developed an e-learning package to educate our workforce.

